# Factors associated with post-bronchoaspiration survival: A cross-sectional study^☆^^☆☆^

**DOI:** 10.1016/j.bjorl.2025.101611

**Published:** 2025-05-27

**Authors:** Cristina Zerbinati Carro, Francisco Winter dos Santos Figueiredo, Luiz Vinicius de Alcantara Sousa, Fernando Adami, Flávio Carneiro Hojaij

**Affiliations:** a Faculdade de Medicina do ABC, Laboratório de Epidemiologia e Análise de Dados, Santo André, SP, Brazil; b Universidade de São Paulo (USP), Faculdade de Medicina (FM), São Paulo, SP, Brazil

**Keywords:** Respiratory aspiration, Dysphagia, Aspiration pneumonia, Latrogenic disease

## Abstract

**Objective:**

To analyze the survival rate of adult patients who underwent bronchoaspiration while hospitalized in a public university hospital with oncology care characteristics.

**Methods:**

A 12-month retrospective longitudinal study was carried out using bronchoaspiration risk management and event notification analysis forms filled out in the medical records of patients admitted to this hospital.

**Results:**

The 34 patients who presented the adverse event of bronchoaspiration had their survival rate reduced by 30% in the first month, and only 29.6% of them survived the second month post-event. Women were more vulnerable to clinical complications originating from the general health status decline as well as to acute pulmonary complications arising from sepsis, consequently presenting a greater reduction in survival.

**Conclusion:**

Bronchoaspiration events corroborate an abrupt decrease in patient survival.

**Level of evidence:**

Level III.

## Highlights

• Those who suffered bronchoaspiration had their survival rate reduced by 30%30 % in the first month, and only 29.6% of them survived the second month post-event.
• Women were more vulnerable to clinical complications originating from the general health status decline as well as to acute pulmonary complications arising from sepsis, consequently presenting a greater reduction in survival.

## Introduction

Previously healthy people become physically and immunologically fragile when they are hospitalized. Approximately 90% of hospitalized patients undergo laryngotracheal aspiration at least once during their hospital stay.^1^

From the pathophysiological standpoint, inhalation of oropharyngeal or gastric contents produces an inflammatory response, which can affect both the airways and the lung parenchyma, resulting in acute lung injury, increased hospitalization rate, and worse outcomes in critically ill patients. Reduction in pulmonary compliance and tissue loss due to neutrophilic inflammatory response, decreased alveolar-capillary permeability, and edema formation can lead to hypoxemia and respiratory failure.^2^

The risk factors that cause bronchoaspiration events are well established in the hospital literature, which enables the development of risk management for this condition based on the prevention of these factors. However, data on the survival rate of post-bronchoaspiration patients are scarce.^3^

Thus, what would be the perspective of patient survival after a bronchoaspiration event? This study aimed to analyze the survival rate of adult patients who have undergone massive bronchoaspiration while hospitalized in a public university hospital with oncology care characteristics.

## Methods

This is a 12-month retrospective longitudinal study carried out using bronchoaspiration risk management and event notification analysis forms filled out in the medical records of patients of both sexes aged ≥18 years admitted to a public university hospital with oncology care characteristics and who presented macroaspiration episodes. Pediatric patients and adult patients who did not undergo bronchoaspiration were excluded from the study.

The study was approved by the Research Ethics Committee of the aforementioned institution under protocol CAAE nº 51897415.7.0000.0082. In 12 months, 4,969 people were hospitalized in that institution, and 34 of them had an adverse event of bronchoaspiration with macroaspiration characteristics. Thus, the sample size was defined by the total number of events that occurred during this period and all of them were considered in the analysis.

The study methodology followed the STrengthening the Reporting of OBservational studies in Epidemiology (STROBE) guidelines.^4^, ^5^

Quantitative variables were presented as central tendency and dispersion measures (median and 25th‒75th percentiles, respectively). Qualitative variables were expressed as absolute and relative frequency measures.

Kaplan-Meyer curves were used to describe the overall survival of patients who presented a bronchoaspiration event. Differences in the medians of overall survival according to sample characteristics were analyzed using the Logrank test. A significance level of 5% (p < 0.05) was adopted for all statistical analyses and the data were processed using the STATA® 11.0 software (StataCorp, LLC).

## Results

Thirty-four (34) patients who presented a macroaspiration event were assessed. Most participants were male (73.5%, n = 25), aged >60 years (70.6%, n = 24), admitted to the hospital wards (61.8%, n = 21), did not present a risk of bronchoaspiration at the time of hospital addition (67.7%, n = 23), but ended up developing this risk during hospitalization (87.0%, n = 20) (Table 1).

**Table 1:** Characteristics of adult patients who underwent bronchoaspiration during their stay in a high-complexity hospital.

Variables	n	%
Sex		
Male	25	73.5
Female	9	26.5
Age group		
≤60 years	10	29.4
>60 years	24	70.6
Sector		
Ward	21	61.8
ICU	13	38.2
Risk		
Without	23	67.7
With	11	32.3
Developed risk		
No	3	13.0
Yes	20	87.0
Deaths		
No	13	38.24
Yes	21	61.76

ICU, Intensive Care Unit.

**Fig. 1:**
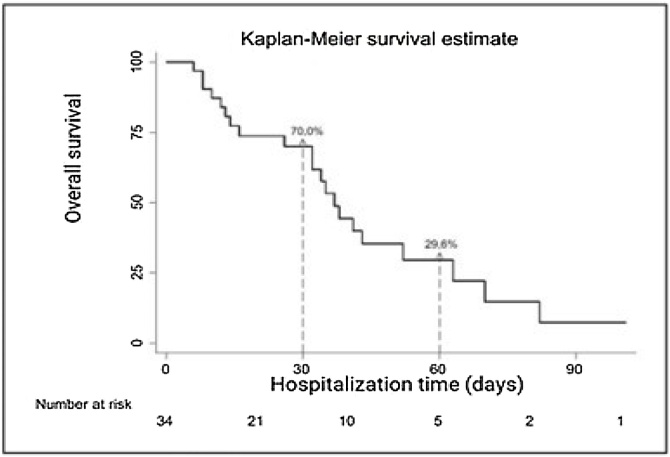
Survival of patients after bronchoaspiration with macroaspiration characteristics.

Fig. 1 shows the overall survival of the studied patients. The 34 patients who presented the adverse event of bronchoaspiration had their survival rate reduced by 30% in the first month, and only 29.6% of them survived the second month post-event (Fig. 1).

Sex was the only related factor among those that could interfere with the survival of these patients (Logrank test, p = 0.008), with a median survival rate of men higher than that of women (Table 2).

**Table 2:** Factors associated with a median survival rate of post-bronchoaspiration patients in a hospital with oncology care characteristics.

Characteristics	Median survival rate (in days)	p^c^
Sex		
Male	52	0.008
Female	26	
Age group		
≤60 years	43	0.635
>60 years	35	
Sector		
Ward	35	0.485
ICU	37	
Risk of bronchoaspiration^a^		
No	41	0.425
Yes	35	
Developed risk^b^		
No	38	0.552
Yes	52	

ICU, Intensive Care Unit.

a Patients admitted at risk of bronchoaspiration.

b Patients who developed the risk of bronchoaspiration after hospital admission.

c Logrank test.

**Fig. 2:**
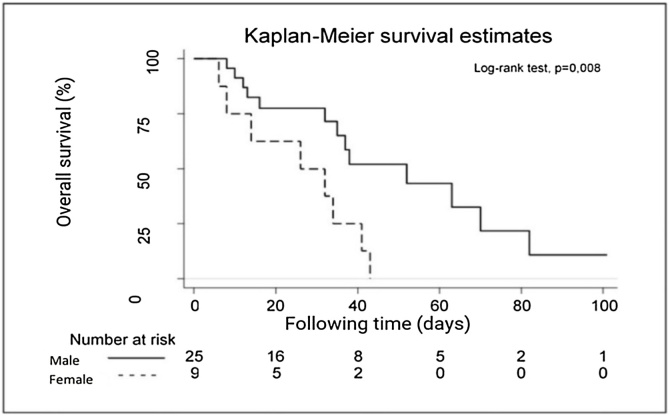
Median post-macroaspiration survival rate by sex in a public university hospital with oncology care characteristics.

Analysis of the median survival rate of these patients according to sex shows that, from the 10th post-bronchoaspiration day, the survival of male patients is greater than that of female patients (Fig. 2).

## Discussion

The main results found in the analysis of factors associated with post- bronchoaspiration survival were: I) There is a rapid and progressive decrease in survival; II) The median survival rate of men is higher than that of women.

Limitations of this study include the fact that it was not performed in a multicenter and with a larger sample size. An information bias related to the completion of the medical records with possible underreporting of events should also be considered. However, in addition to being one of the few studies addressing the survival rate of post-bronchoaspiration patients, it covered a heterogeneous age group and observed characteristics that can affect any population.

For years, the national and international literature has discussed macroaspirations, which have been studied by large research groups, as in this study. Macroaspirations are identified by the health team based on manifestations of greater magnitude and evidence of clinical changes. It has also sometimes been questioned in the clinical field whether there is an infectious or inflammatory impact on the lower airways, the so-called chemical pneumonia or pneumonitis, respectively.^3^, ^6^, ^7^, ^8^

Another aspect often discussed regards the fact that these events can be avoided with the application of daily care measures. Macroaspirations are adverse events that can be minimized in about 90% of cases through daily and patient-centered care, with rapid risk identification and active prevention implementation. Nevertheless, it is also known that there is a possibility that 10% of cases present aspirations due to comorbidities and worsening of the clinical condition, especially of the gastrointestinal tract.^9^

In parallel, there is still an issue little discussed in the clinical and research fields, which is the dimension of clinical complications inherent in the aspiration process.^10^ Although this study was conducted with a focus on macroaspirations, it showed that the death outcome occurs rapidly, suggesting that bronchoaspiration is a harmful adverse event to the health of patients. Corroborating these findings, in 2022, Sanivarapu and Gibson^11^ 11 reported that the mortality rate due to aspiration pneumonia can reach 70% of cases and added that it is also associated with and dependent on the volume and content aspirated.

Also in 2022, a case-control study conducted in the United States^7^ investigated the incidence, trends, and risk factors regarding mortality from aspiration pneumonia and found that, in this population, neurological, gastrointestinal, and pulmonary changes in advanced age were associated with death outcomes. They also mentioned that aspiration pneumonia was the underlying cause of tens of thousands of deaths each year in that country, once again suggesting that this event presents unfavorable patient outcomes, as observed in this study.

Current studies in the national and international literature are unanimous in describing that these outcomes are not favorable to patients, and most of them mention the risk of high lethality, although not measured, as in the present study. This study showed in time how lethal this event was, with a high death rate within 60 days post-event.

Males had a higher survival rate than females. From this finding, the possibility of women presenting more comorbidities or greater pathological impairment was discussed, contributing to advances in frailty compared to men. This information deserves further investigation and other studies on this theme can corroborate this reasoning.

## Conclusion

Bronchoaspiration events corroborate an abrupt decrease in patient survival.

## Declaration of competing interest

The authors declare no conflicts of interest.
